# Chemoinformatic Database Building and in Silico Hit-Identification of Potential Multi-Targeting Bioactive Compounds Extracted from Mushroom Species

**DOI:** 10.3390/molecules22091571

**Published:** 2017-09-19

**Authors:** Annalisa Maruca, Federica Moraca, Roberta Rocca, Fulvia Molisani, Francesca Alcaro, Maria Concetta Gidaro, Stefano Alcaro, Giosuè Costa, Francesco Ortuso

**Affiliations:** Laboratorio di Chimica Farmaceutica, Dipartimento di Scienze della Salute, Università “Magna Græcia” di Catanzaro, Viale Europa, 88100 Catanzaro, Italy; maruca@unicz.it (A.M.); fmoraca@unicz.it (F.Mor.); rocca@unicz.it (R.R.); fulviamolisani@gmail.com (F.Mol.); francesca.alcaro@gmail.com (F.A.); mgidaro@unicz.it (M.C.G.); gcosta@unicz.it (G.C.); ortuso@unicz.it (F.O.)

**Keywords:** database, mushrooms, polypharmacology, multi-target, virtual screening, metabolic diseases, anti-cancer, neurodegenerative diseases, anti-inflammatory

## Abstract

Mushrooms are widely-consumed fungi which contain natural compounds that can be used both for their nutritive and medicinal properties, i.e., taking advantage of their antimicrobial, antiviral, antitumor, anti-allergic, immunomodulation, anti-inflammatory, anti-atherogenic, hypoglycemic, hepatoprotective and antioxidant effects. Currently, scientific interest in natural compounds extracted from the fungal species is increasing because these compounds are also known to have pharmacological/biological activity. Unfortunately, however, their mechanisms of action are often unknown, not well understood or have not been investigated in their entirety. Given the poly-pharmacological properties of bioactive fungal compounds, it was decided to carry out a multi-targeted approach to predict possible interactions occurring among bioactive natural fungal extracts and several macromolecular targets that are therapeutically interesting, i.e., proteins, enzymes and nucleic acids. A chemical database of compounds extracted from both edible and no-edible mushrooms was created. This database was virtually screened against 43 macromolecular targets downloaded from the Protein Data Bank website. The aim of this work is to provide a molecular description of the main interactions involving ligand/multi-target recognition in order to understand the polypharmacological profile of the most interesting fungal extracts and to suggest a design strategy of new multi-target agents.

## 1. Introduction

The term “mushroom” does not refer to a taxonomic category. According to the definition of Chang and Miles, “mushrooms are macrofungi with distinctive fruiting bodies that could be hypogenous or epigenous, large enough to be seen by naked eyes and to be picked by hands” [1]. From a taxonomic point of view, mushrooms are mainly represented by basidiomycetes and some species of ascomycetes. Although the number of mushroom species distributed on Earth is estimated to be 140 K, only 22 K are known and approximately 7000 have the potential to benefit humanity [2]. Several studies have shown that mushrooms possess various bioactivities (mainly, but not limited to) antioxidant, anti-inflammatory, anticancer, immunomodulatory, antimicrobial, hepatoprotective, and antidiabetic properties. Therefore, it appears that mushrooms could be considered a functional food that can be consumed to prevent and treat several chronic diseases, such as cancer, diabetes mellitus and neurodegenerative disorders [3]. In fact, multiple health benefits have been recognized for some mushroom species (*Ganoderma lucidum*, *Inonotus obliquus*, *Lentinula edodes*, *Agaricus bisporus*, *Fomitopsis pinicola*), which exhibit potent medicinal and functional properties. Mushrooms have also been found to be rich in vitamins (i.e., thiamine, riboflavin, niacin, biotin, ascorbic acid), lipids (mono-, di-, and triglycerides, sterols, phospholipids), proteins/glycoproteins, food fibers, polysaccharides (i.e., β-glucans), nucleic acid derivatives (hypocholesterolemic eritadenine) and peptides [4]. Due to their metabolism, higher fungi can produce an overabundance of secondary functional metabolites including but not limited to polyketides, terpenes, steroids and phenolic compounds with important beneficial properties thereby providing more opportunity to discover new lead compounds [5]. Most of the mushrooms’ secondary metabolites possess drug-like structures (i.e., Lipinski’s Rule of Five compliant) and could be considered a major natural inspiration for drug discovery purposes [6]. Mushrooms also represent a source of polysaccharides with anticancer and immunostimulating properties. Polysaccharides found in mushrooms do not directly attack cancer cells, but rather produce antitumor effects by activating an immune response in the host [7]. Indeed, the polysaccharide fraction of the *Agaricus bisporus (A. bisporus)* extract, detected in a variety of different mushroom species, showed immunostimulatory and antitumor activities [8]. The edible mushroom *Hericium erinaceus (H. erinaceus)* contains valuable constituents such as polysaccharides, lectins, proteins, lipids, hericenone, erinacol, erinacine, and terpenoids that possess known antitumor activity [9]. The ethanol extract of the species *Fomitopsis pinicola* (*F. pinicola*) induces cancer cell apoptosis both in vitro and in vivo [10]. Phenols and other phytoconstituents found in *Ganoderma lucidum* (*G. lucidum*) have antioxidant and chelating properties [11]. Polysaccharides (in particular Ganoderan, a β-d-glucan), proteins and triterpenoids have been isolated from *G. lucidum* having strong immuno-modulating action and a hepatoprotective activity. Ganoderan increases the expression of a major histocompatibility complex (MHC) class II molecule on antigen-presenting macrophages. Furthermore, there is other evidence that suggests extracts from *G. lucidum* can modulate humoral or B-cell mediated immunity [12]. Its triterpenoids have powerful protective effects against liver damage. This hepatoprotective effect is related to its ability to increase the activity of free radical scavenging enzymes and to be a potent inhibitor of β-glucuronidase activity which is an indicator of hepatic damage [13]. The polysaccharides extracted from the mushroom *Inonotus obliquus (I. obliquus)* revealed anti-hyperglycemic effects due to a significant decrease in blood glucose levels [3]. Debnath and co-workers also demonstrated that ethanol extracted from *I. obliquus* has remarkable antioxidant and anti-inflammatory activities suppressing pro-inflammatory mediators such as NO, PGE2, iNOS, COX-2, TNF-α, IL-1β, and IL-6 [14]. Anti-inflammatory activity is an essential property of bioactive mushrooms to promote positive effects on health. Cordymin, a peptide purified from *Cordyceps sinensis (C. sinensis)*, has a neuroprotective effect in the ischemic brain attributed to the inhibition of inflammation and enhancement of antioxidant activity related to lesion pathogenesis [15]. The mycelia and fruiting body extracts of numerous mushrooms show antimicrobial activity against a wide range of infectious microorganisms. A number of antimicrobial agents isolated from mushrooms may prove themselves beneficial for human health. The antimicrobial effects of the crude extracts of the mushroom *Auricularia auricular-judae* showed antibacterial effects towards *Escherichia coli* and *Staphylococcus aureus* [16]. *Lentinula edodes* (*L. edodes*) contains oxalic acid, which is responsible for antimicrobial effects against *Staphylococcus aereus* and other bacteria [17]. Lentinan, a pure polysaccharide, is the first compound isolated from *L. edodes* that has shown antitumor and ant proliferative effects [18,19]. Lentinan has been proven to prolong the survival rates of cancer patients, in particular with gastric or colorectal cancer [20,21,22]. Lentinan increases the host’s resistance against various forms of cancer and is able to restore the immune function of affected individuals. It can inhibit prostaglandin synthesis slowing down T cell differentiation [23]; it increases the activation of nonspecific inflammatory responses [24] and enhances vasodilation [25]. Proteins, peptides, polysaccharides, lipopolysaccharides, glycoproteins, and lipid derivatives have potent effects on the immune system [22]. In addition, different studies have shown that mushrooms have antiviral [26], antiallergic [27,28], antitubercular [29], antifungal [30] activities and hypoglycemic effects [31]. Finally, the compounds extracted from *Pleurotus giganteus*, *Lignosus rhinocerotis* and *Grifola frondosa*, reduced beta amyloid-induced neurotoxicity, anti-acetylcholinesterase, neurite outgrowth stimulation, nerve growth factor (NGF) synthesis, neuroprotective, antioxidant, and anti-neuroinflammatory effects [32]. In the opinion of Chang, mushrooms compounds are the “wave of the future” because they can boost the body’s defense mechanism [33]. However, their role in human health is still largely unexplored. The current aim is to comprehensively evaluate the bioactivities of some underutilized mushrooms. Special attention should be paid to the mechanisms of action of the active components. Herein, the intention is to extend the knowledge of the proteins, enzymes and nucleic acids involved as targets in complicated diseases such as cancer, inflammation, neurodegenerative diseases and metabolic diseases. This will allow the possible prediction of interactions between several compounds extracted from mushrooms. Today, the polypharmacological concept is gaining significant importance especially for treating complicated diseases. Multi-target drugs have different advantages compared to single target drug co-administration mainly higher efficacy, an improved safety profile and better compliance. Furthermore, virtual screening methods help identify sources of off-target drug effects and to investigate their potential to cause adverse or desirable side effects [34]. In this work, a database of compounds has been created from the extracts of 162 mushroom species (both edible and toxic). Furthermore, docking studies were carried out to give a theoretical comprehension of their polypharmacological activity and to guide the design of promising new multi-target agents (MTAs).

## 2. Results and Discussion

The Protein Data Bank (PDB) [35] models used for the virtual screening were divided into four categories based on their therapeutic implications: (a) targets involved in cancer, (b) neuronal degenerative diseases, (c) inflammatory and (d) metabolic diseases. Moreover, in order to give a detailed picture of the most important interactions, re-docking experiments were performed for each PDB model with the relative co-crystallized ligand (see Supplementary Material and Table S1) and the results compared with the best MTAs docking.

### 2.1. Multi-Targeted Anticancer Compounds

Most of the conventional chemotherapeutic agents used today were designed to hit a single target. However, the physiological and mechanistic deregulations responsible for cancer implicate the involvement of different genes or signaling cascades. In these cases, a multi-target approach might be required to achieve optimal therapeutic benefit [36]. Table 1 lists the fungal chemical compounds with the best theoretical affinities (G-score) towards most of the anticancer targets.

**Figure 1:**
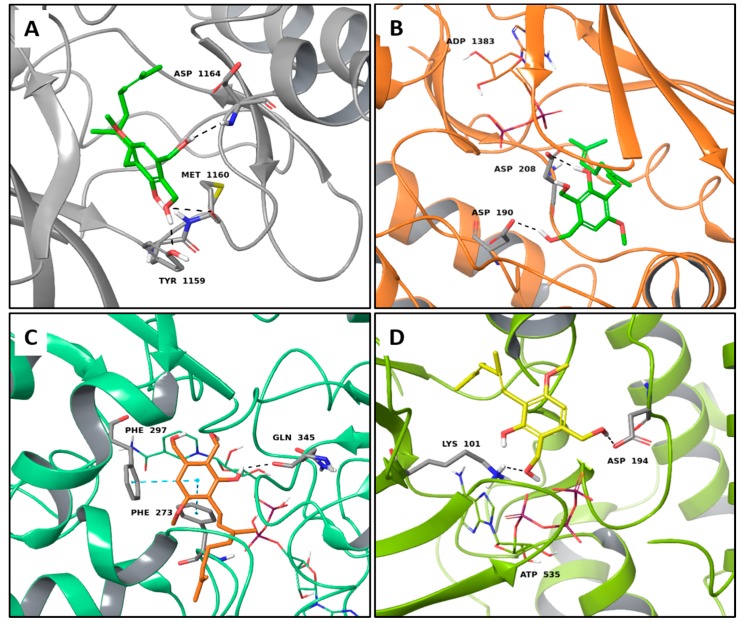
Molecular interactions of hericenol A and anticancer targets: (**A**) c-Met protein (PDB: 2WGJ). Protein is shown as a gray cartoon while ligand is shown as green carbon sticks; (**B**) MEK1 protein (PDB: 4ARK). Protein is shown as an orange cartoon while ligand is shown as green carbon sticks; (**C**) SIRT1 target (PDB: 4I5I). Protein is shown an aquamarine cartoon while ligand is shown as orange carbon sticks; (**D**) MEK2 protein (PDB: 1S9I). Protein is shown as a light green cartoon while ligand is shown as yellow carbon sticks. H-bonds and π-π interactions are shown as dashed black and cyan lines, respectively. Amino acid residues involved in the molecular interactions are shown as gray sticks.

The edible mushroom *H. erinaceus* contains valuable constituents and has demonstrated antitumor activity [9]. As noted from Table 1, compounds extracted from *H. erinaceus* (hericerol A and erinacerin P) have good theoretical binding affinity versus four and two anticancer targets, respectively. Specifically, the best binding affinity of the phenolic derivative hericenol A occurs with Mesenchymal-Epithelial Transition Factor (c-Met) kinase (Table 1). Such an observation can be attributed to its three hydrogen bonds (H-bonds) with the Asp1164, Met1160 and Tyr1159 residues (Figure 1A), while the establishment of only two H-bonds with Asp208 and Asp190 is responsible for the highest binding affinity against the Mitogen-activated protein kinase 1 (MEK1) protein (Figure 1B). The presence of two T-shaped π-π interactions between the phenolic ring of hericenol A and the Phe297 and Phe273 and one H-bond with Gln345 residues of NAD-dependent protein deacetylase sirtuin-1 (SIRT1) are instead essential for improving the binding affinity (Figure 1C). Finally, hericenol A establishes only two H-bonds with Asp194 and Lys101 of Mitogen-activated protein kinase 2 (MEK2, Figure 1D).

**Figure 2:**
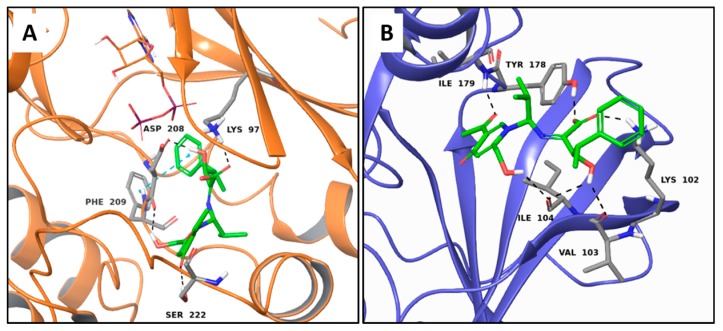
Molecular interactions of erinacerin P and the anticancer targets: (**A**) Protein target, MEK1 (PDB: 4ARK). Protein is shown as an orange cartoon; (**B**) Protein target, SGK1 (PDB: 3HDM). Protein is shown as violet cartoon. Erinacerin P is represented as green carbon sticks. H-bonds and π-π interactions are displayed as dashed black and cyan lines, respectively. Amino acid residues involved in the molecular interactions are shown as gray sticks.

Erinacerin P establishes three H-bonds to Asp209, Lys97 and Ser 222, and one π-π interaction to Phe209 in MEK1 (Figure 2A). Six H-bonds to Val103, Ile104, Lys102, Ile179 and Tyr178 are responsible of the good theoretical binding affinity toward serine/threonine-protein kinase 1 (SGK1, Figure 2B).

**Figure 3:**
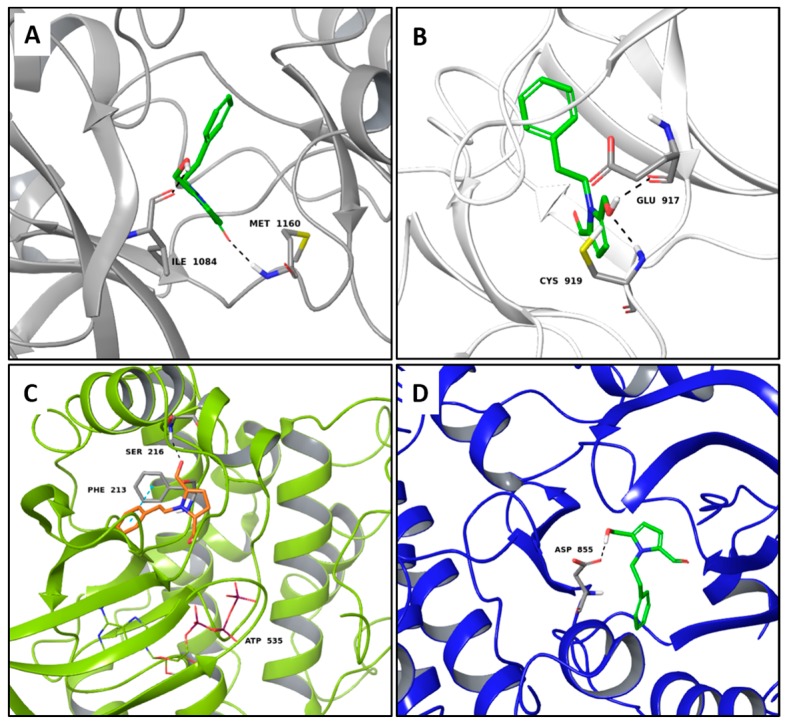
Molecular interactions of inotopyrrole and the anticancer targets: (**A**) Protein target, c-Met (PDB: 2WGJ). Protein is shown as a gray cartoon while ligand is shown as green carbon sticks; (**B**) Protein target, VEGFR2 (PDB: 3VHE). Protein is shown as a white cartoon while ligand is shown as green carbon sticks; (**C**) Protein target MEK2 (PDB: 3POZ). Protein is shown as a light green cartoon while ligand is shown as orange sticks; (**D**) Protein target EGFR. Protein is shown as a blue cartoon while ligand is shown as green sticks. H-bonds s and π-π interactions are shown as dashed black and cyan lines, respectively. Amino acid residues involved in the interactions are shown as gray sticks.

Inotopyrrole, an alkaloid pyrrole isolated from mycelium of *Inonotus obliquus*, shows a satisfactory theoretical binding affinity relative to c-Met kinase and vascular endothelial growth factor receptor 2 (VEGFR2, by establishing two H-bonds in both complexes. In particular, the amino acid residues involved in the stabilization of the complexes are Ile1084 and Met1160 of c-Met and Cys919 and Glu917 of VEGFR2 (Figure 3A,B). On the other hand, in the binding pocket of MEK2, inotopyrrole is only able to form one H-bond and one π-π interaction with Ser216 and Phe213, respectively (Figure 3C). Finally, in the stabilization of the complex between the alkaloid and the epidermal growth factor receptor (EGFR) target, only one H-bond is present, bringing the carboxyl group of Asp855 and the hydroxyl group of the ligand closer (Figure 3D).

**Figure 4:**
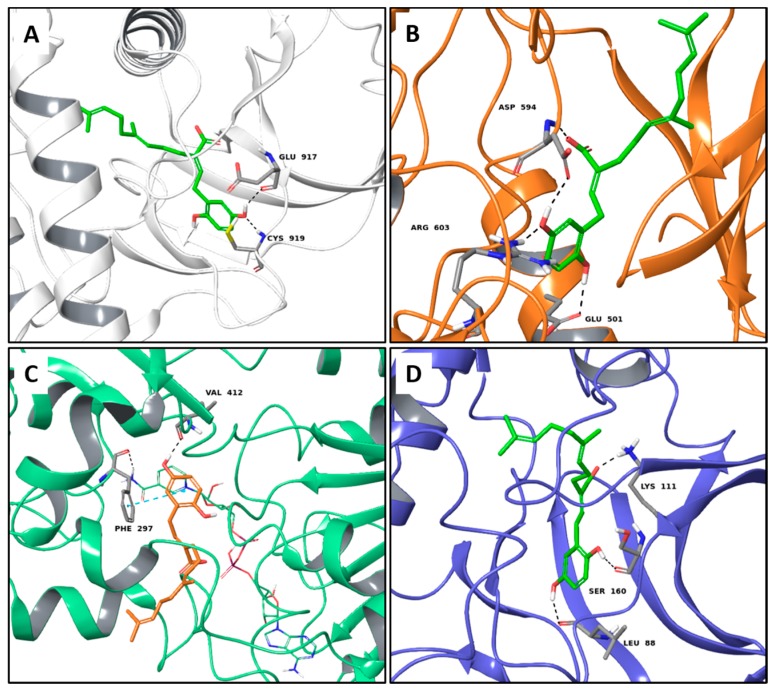
Molecular interactions of ganomycin B and the anticancer targets: (**A**) Protein target, VEGFR2 (PDB: 3VHE). Protein is shown as a white cartoon while ligand is shown as green carbon sticks; (**B**) Protein target, B-RAF V600E (PDB: 3OG7). Protein is shown as an orange cartoon while ligand is shown as green sticks; (**C**) Protein target, SIRT1 (PDB: 4I5I). Protein is shown as an Aquamarine cartoon while ligand is shown as orange carbon sticks; (**D**) Protein target, PDK1 (PDB: 3NAX). Protein is shown as a light blue cartoon while ligand is shown as orange carbon sticks. H-bonds and π-π interactions are shown as dashed black and cyan lines, respectively. Amino acid residues involved in the interactions are shown as gray sticks.

Amongst the mushrooms containing compounds with anti-cancer and antioxidant activities, we also found *G. lucidum* [11], from which the farnesyl hydroquinone ganomycin B was extracted. It specifically recognizes four targets involved in cancer pathology such as VEGFR2, B-RAF V600E (B-Raf proto-oncogene serine/threonine-protein kinase V600E), SIRT1 and phosphoinositide-dependent kinase 1 (PDK1). In particular, its best G-Score, was predicted for the complex with PDK1, establishing three H-bonds with the backbone of Leu88, Ser160 and the sidechain of Lys111 (Figure 4D). Its interactions against VEGFR2 are based on two H-bonds with the backbone portion of Glu917 and Cys919 (Figure 4A), while in the binding pocket of B-RAF V600E, it forms four H-bonds to Asp594, Arg603, and Glu501 (Figure 4B). Finally, the formation of one H-bond with Val412 and one π-π interaction to Phe297 characterize its complex with SIRT1 (Figure 4C).

When comparing the docking results of the best MTAs of the mushroom extracts with those of the co-crystallized ligand (Figure S1 and Table S1 above) in most cases, the results show that at least one interaction is shared between the co-crystallized ligand and the fungal chemical components having the best theoretical affinities. For example, both hericenol A, inotopyrrole and the co-crystallyzed ligand of c-Met, crizotinib, (Figure 1A, Figure 3A and Figure S1A, respectively) share an H-bond with the residue Met1160. Likewise, a very similar binding interaction is observed between erinacerin P and the inhibitor, complexed with the anticancer target MEK1 (PDB code: 4ARK) (Figure 2A and Figure S1B, respectively), with both their aromatic rings involved in one π-π interaction with Phe209, and their side chains forming two H-bonds with Lys97 and Asp208. The formation of two H-bonds with Lys111 and Ser160 is shared between ganomycin B and the inhibitor complexed with PDK1 (PDB code: 3NAX) (Figure 4D and Figure S1I, respectively).

### 2.2. Multi-Targeted Anti-Inflammatory Compounds

Inflammation is an immune system response to fight a harmful external stimulus often caused by bacteria, viruses or other pathogens. However, inflammation is not always a helpful response for the body. In several chronic diseases such as rheumatoid arthritis, osteoarthritis, atherosclerosis, diabetes, neurodegeneration, allergies and infections, the immune system fights against its own cells thereby causing harmful inflammatory responses. In such cases, the development of anti-inflammatory multi-target drugs can be an extremely helpful [37]. A global amount of six inflammation-involved targets were used in this study (Table 2). Compounds exhibiting the best affinity to multiple targets involved in inflammation are reported in Table 2.

**Figure 5:**
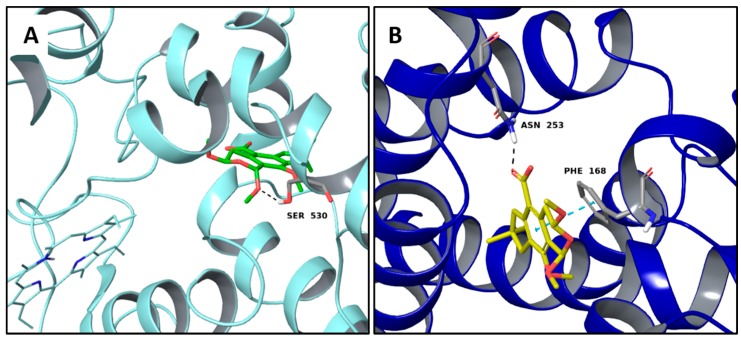
Molecular interactions of illudacetalic Acid and two anti-inflammatory targets: (**A**) Protein target, COX-2 (PDB: 5IKR). Protein is shown as a turquoise cartoon while ligand is shown as green carbon sticks; (**B**) Protein target, adenosine A2A receptor (PDB: 3RFM). Protein is shown as a dark blue cartoon while ligand is shown as yellow carbon sticks. H-bonds and π-π interactions are shown as dashed black and cyan lines respectively. Amino acid residues involved in the interactions are shown as gray sticks.

Illudacetalic acid and pterulinic acid, extracted from *Omphalotus olearius*, have shown good theoretical binding affinity against relevant targets involved in inflammations. Illudacetalic acid recognizes well the cyclooxygenase 2 (COX-2) binding pocket by means of one H-bond to Ser530 (Figure 5A), also shared by the PDB complexed ligand mefenamic Acid (Figure S2A).

Moreover, the complex with the adenosine A2A receptor is characterized by one H-bond between the ligand carboxyl group and the side chains of Asn253 that is also involved in one H-bond with its complexed inhibitor (Figure S2B), while a π-π interaction to Phe168 involves the ligand aromatic moiety (Figure 5B).

**Figure 6:**
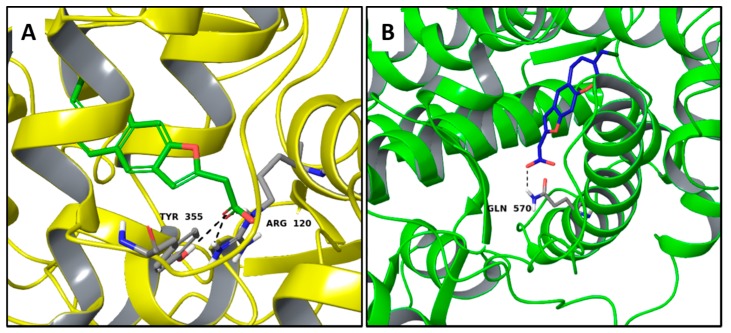
Molecular interactions of pterulinic Acid and two anti-inflammatory targets: (**A**) Protein target, COX-1 (PDB: 1Q4G). Protein is shown as a yellow cartoon while ligand is shown as green carbon sticks; (**B**) Protein target: glucocorticoid receptor (PDB: 1M2Z). Protein is shown as a florescent green cartoon while ligand is shown as dark blue carbon sticks. H-bonds and π-π interactions are shown as dashed black and cyan lines respectively. Amino acid residues involved in the interactions are shown as gray sticks.

Regarding pterulinic acid, it is a novel halogenated antibiotic which shows significant antifungal and weak or no cytotoxic activities since it is an inhibitor of eukaryotic respiration by means of binding to mitochondrial NADH: ubiquinone oxidoreductase [38]. In the present work, a satisfactory theoretical binding affinity was found between this compound, cyclooxygenase 1 (COX-1) and the glucocorticoid receptor. Particularly, the complexes are stabilized by H-bonds via Tyr530 and Arg120 in COX-1 and with Gln570 in the glucocorticoid receptor (Figure 6A,B). For the COX-1 target, it should be noted that pterulinic Acid binds similarly to the complexed inhibitor biphenylacetic acid (Figure S2C) also sharing the two H-bonds with Tyr530 and Arg120.

### 2.3. Multi-Targeted Compounds in Neurodegenerative Disease

Despite being characterized by specific molecular mechanisms and clinical evidence, some neurodegeneraive disorders such as Parkinson’s (PD), Alzheimer’s (AD) and Huntington’s diseases (HD), together with the amyotrophic lateral sclerosis (ALS), share a multifactorial origin. Consequently, multi-target strategies have increasingly been taken into consideration for the treatment of such chronic diseases [39]. Several MTAs have already been designed for their potential application against some of the mentioned neurodegenerative diseases [40]. For example, lipocrine was designed to inhibit the catalytic activity of AChE and also to protect against ROS [41] thereby bringing benefits in Alzheimer’s disease. Similarly, caproctamine reports AChE inhibitor and competitive muscarinic M_2_ receptor antagonist structural features, showing a multi-target profile for the treatment of this disorder [42]. Also some natural compounds hold multiple functions. Curcumin can be considered a promising candidate to prevent AD, due to well-known antioxidant, anti-inflammatory activities and *anti-amyloidogenic effects* [43]. In the current study, the most interesting fungal-extract compounds for neurodegenerative targets are reported in Table 3.

**Figure 7:**
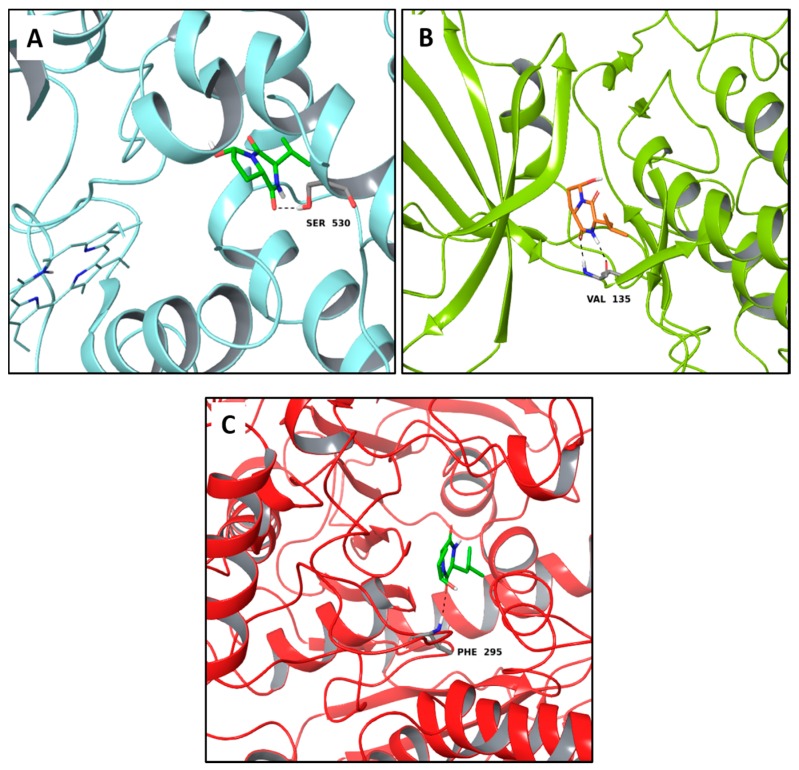
Molecular interactions of cordysinin A and three targets involved in the neurodegenerative diseases: (**A**) Protein target, COX-2 (PDB: 5IKR). Protein is shown as a turquoise cartoon while ligand is shown as green carbon sticks; (**B**) Protein target, GSK3β (PDB: 4ACC). Protein is shown as a light green cartoon while the ligand is shown as orange carbon sticks; (**C**) Protein target: AChE (PDB: 4EY7). Protein is shown as a red cartoon while ligand is represented as green carbon sticks. H-bonds are shown as dashed black. Amino acid residues involved in the interactions are shown as gray sticks.

Interestingly, among the compounds reported in Table 3, cordysinin A is contained in *Cordyceps sinensis*, whose extracts are reported in the literature for their neuroprotective effects [15], particularly addressing inflammation inhibition and the antioxidant activity. According to this information, cordysinin A shows a good theoretical binding affinity relatively to glycogen synthase kinase-3 beta (GSK3β), acetylcholinesterase (AChE) and COX-2, by establishing H-bonds with the residues Val130, Phe295 and Ser530, respectively (Figure 7A–C).

Interestingly, pterulone resulted as the most promising multi-target compound especially since its theoretical affinity profile suggests concurrent activity against four neurodegenerative disorders involved targets: AChE, monoamine oxidases B (MAO-B), COX-2 and GSK3β. Pterulone is a fungal metabolite derived from *Omphalotus olearius* and is reported in the literature together with pterulinic acid as a novel halogenated antibiotic [38].

**Figure 8:**
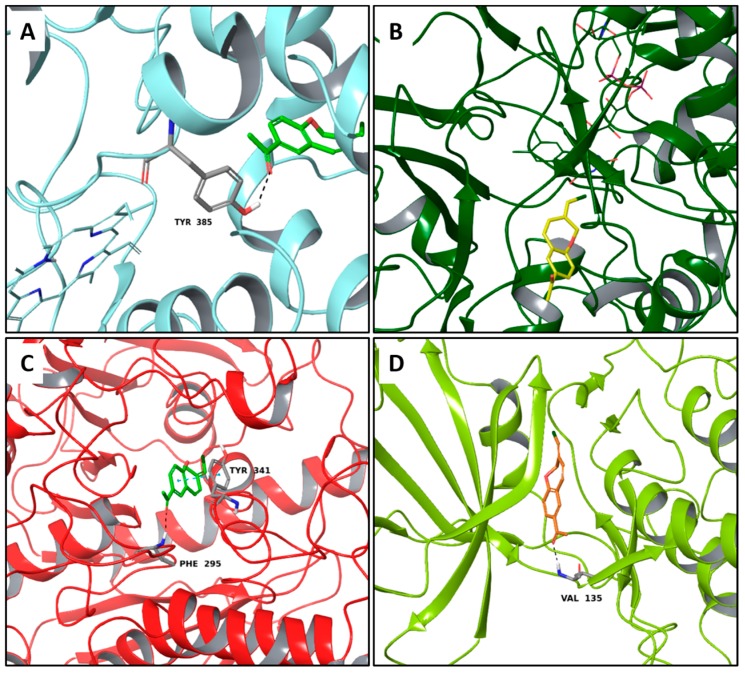
Molecular interactions of Pterulone and its four targets involved in the neurodegenerative diseases: (**A**) Protein target, COX-2 (PDB: 5IKR). Protein is shown as a turquoise cartoon while ligand is shown as green carbon sticks; (**B**) Protein target: MAO-B (PDB: 2V5Z). Protein is shown as a dark green cartoon while ligand is shown as yellow carbon sticks; (**C**) Protein target, AChE (PDB: 4EY7). Protein is shown as a red cartoon while ligand is shown as green carbon sticks; (**D**) Protein target, GSK3β (PDB: 4ACC). Protein is shown as a light green cartoon while ligand is shown as orange carbon sticks. H-bonds and π-π interactions are shown as dashed black and cyan lines, respectively. Aminoacidic residues involved in the interactions are shown as gray sticks.

In best poses within COX-2 and GSK3β, it establishes one H-bond to Tyr385 and to Val135, respectively (Figure 8A–D). Its complex with AChE is stabilized by one H-bond to Phe295 and a π-π interaction to Tyr341 (Figure 8C). Conversely, in the binding pocket of MAO-B enzyme, the recognition of pterulone is based only on hydrophobic contributions (Figure 8B). It is worth pointing out that in the abovementioned targets, both cordysinin A and pterulone also share the same complexed ligand interactions. For example like donepezil, AChE, cordysinin A and pterulone form one H-bond with the Phe295 residue (Figure 7C, Figure 8C and Figure S3B), while in the case of GSK3β, they share the interaction with Val135 (Figure 7B and Figure 8D) with respect to the co-crystallized ligand (Figure S3B).

### 2.4. Multi-Targeted Compounds in Metabolic Diseases

Metabolic diseases such as the type 2 diabetes mellitus and hyperlipidemia, are coupled to high risk complications related to cardiovascular and coronary diseases as well as diabetic nephropathy, retinopathy and neuropathy. The rate of death among patients with type 2 diabetes is approximately two times higher when compared to people without the disorder. Furthermore, clinical trials on single risk factors have shown effectiveness in reducing the development and progression of complications [44]. In particular, the latter are reduced by the administration of a “cocktail of drugs”, which contains several compounds interacting against multiple targets. For this reason, the actual aim is to develop a single compound with multi-target synergistic mechanism of action, in order to limit the side effects caused by the “cocktail of drugs” strategy. Recently, a series of naturally occurring pentacyclic triterpenes have been evaluated as multi-target therapeutic agents for the prevention and the treatment of metabolic and vascular diseases when administered orally showing both effectiveness and very low toxicity [45]. From the virtual screening results, some natural compounds showed a good theoretical affinity with different targets involved in metabolic diseases, particularly in diabetes mellitus (Table 4).

**Figure 9:**
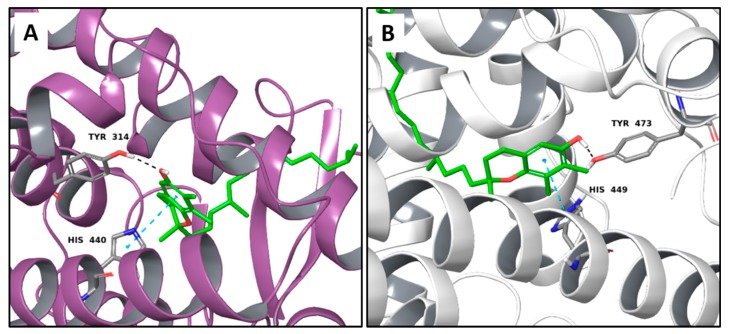
Molecular interactions between O-xylotocopherol and its two best targets involved in the metabolic diseases: (**A**) Protein target PPAR-α (PDB: 3VI8). Protein is shown as a purple cartoon; (**B**) The protein target, PPAR-γ (PDB: 2PRG). Protein is shown as a white cartoon. In both pictures ligand is represented as green carbon sticks. H-bonds and π-π interactions are shown as dashed black and cyan lines respectively. Amino acid residues involved in the interactions are shown as gray sticks.

As reported in the Table 4, O-xylotocopherol is the compound with the best G-Score towards its targets. It belongs to a class of methylated phenols, many of which have vitamin E like activity. This compound was found in different mushrooms, although mostly in *Mycena Rosea* and *Lactarius Salmonicolor.* In the literature [46] it is reported that O-xylotocopherol has the ability to inactivate the most reactive species of oxygen and to protect unsaturated fatty acids from oxidation. O-xylotocopherol shows a good theoretical affinity towards two important targets in metabolic diseases: peroxisome proliferator-activated receptor alpha (PPAR-α) and peroxisome proliferator-activated receptor gamma (PPAR-γ). Interestingly, it shows a similar binding mode in both complexes since it establishes one H-bond and one π-π interaction to Tyr314 and His440 of PPAR-α (Figure 9A) and to Tyr473 and His449 of PPAR-γ (Figure 9B), respectively. Furthermore, His440 and Tyr473 of PPAR-α and PPAR-γ respectively, are also involved in the interactions with the complexed compounds APHM13 and rosiglitazione.

### 2.5. Non Selective MTAs

Analayzing the in silico virtual screening data, it has emerged that some fungal hits are able to non-selectively interact with most of the targets with a good theoretical binding affinity (Table S2 of the Supplementary Materials). These non-selective molecules can be considered as an example of compounds with off-target activity. Indeed, virtual screening methods can be helpful to identify sources of off-target drug effects, investigating their adverse or desirable side effects, obtained as a result of modulation of other targets. Moreover, current methods can enrich the design of off-target pharmacology. In particular, compounds that are rationally designed with a well-defined multitarget profile must be distinct from non-selective (or “dirty”) drugs that often possess irrelevant off-target activities for the studied disease and frequently give rise to deleterious side effects [47].

**Figure 10:**
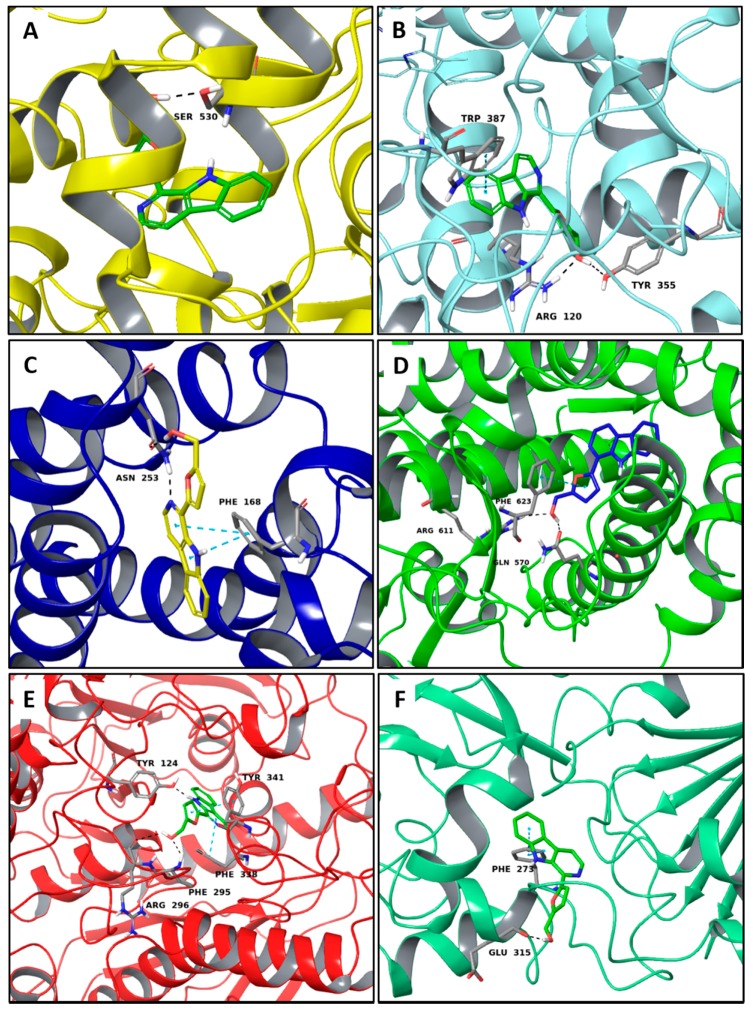
Molecular interactions between perlolyrine, the best non-selective MTA, and its best targets: (**A**) The protein target, COX-1 (PDB: 1Q4G). Protein is shown as a yellow cartoon while ligand is shown as green carbon sticks; (**B**) Protein target: COX-2 (PDB: 5IKR). Protein is shown as a turquoise cartoon while ligand is shown as green carbon sticks; (**C**) Protein target: adenosineA2A receptor (PDB: 3RFM). Protein is shown as a dark blue cartoon while ligand is shown as yellow carbon sticks; (**D**) Protein target: glucocorticoid receptor (PDB: 1M2Z). Protein is shown as a florescent green cartoon while ligand is shown as dark blue carbon sticks; (**E**) Protein target: AChE (PDB: 4EY7). Protein is shown as a red cartoon while ligand is represented as green carbon sticks; (**F**) Protein target: SIRT1 (PDB: 4I5I). Protein is shown as an aquamarine cartoon while ligand is shown as green carbon sticks. H-bonds and π-π interactions are shown as dashed black and cyan lines respectively. Amino acid residues involved in the interactions are shown as gray sticks.

Among our hits, we analyzed perlolyrine, an alkaloid extracted from *Cordyceps sinensis,* as positive example of compounds with off-target activity, since it has a good theoretical binding affinity versus important targets involved in the inflammatory process and in the neurodegenerative diseases. Furthermore, in literature another compound, extracted from *Cordyceps sinensis*, such as cordymin, was reported for its neuroprotective effects, which were addressed to the inhibition of inflammation and to antioxidant activity. Perlolyrine is a β-carboline, reported in literature as a hTRPV1- and hTRPA1-activating compound [48]. Analyzing our data, it is able to establish a good affinity with six different targets such as COX-1, COX-2, adenosineA2A receptor, glucocorticoid receptor, AChE and SIRT1. Comparing the complexes with the two COX isoforms, we observed better interactions in the COX-2, where the ligand engaged two H-bonds to Arg120 and to Tyr355 and one π-π interaction to Trp387 (Figure 10B). Conversely, perlolyrine forms only one H-bond to Ser530 of COX-1 (Figure 10A). When it is complexed by adenosine A2A receptor, this ligand is able to establish two π-π interactions to Phe168 and one H-bonds with Asn253, while in the binding pocket of glucocorticoid receptor it establishes two H-bonds with Arg263 and Gln570 and one π-π interaction with Phe263. The best number of interactions, instead, were observed in the AChE complex, where the alkaloid engages three H-bonds with Arg296, Phe295, Tyr124 and two π-π interactions with Phe338 and Tyr341. Finally, analyzing the docking pose of perlolyrine in the binding pocket of SIRT1, we observed the formation of two H-bonds to Glu315 and to Phe273 and one π-π interaction to Phe273.

## 3. Materials and Methods

### 3.1. Database Building

**Figure 11:**
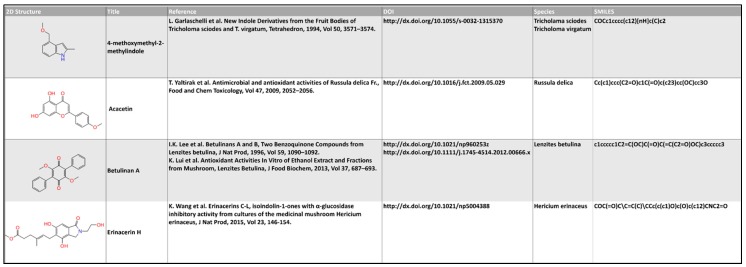
Example of a screenshot in the implementation of the database fragment containing 2D Structure, Title, reference, DOI, species and SMILE of the compounds extracted from the mushrooms.

Data were collected from the available literature at the National Center for Biotechnology Information (NCBI) website [49]. The database of fungal extracts used in the present study contains more than 1103 natural compounds present in 162 different fungal species (Table S3 of the Supplementary Materials). The 2D chemical structures of the identified fungal extracts were downloaded from free access websites such as PubChem [50], ChEMBL [51] and ChemSpider [52]. Missing chemical structures were integrated using MarvinSketch [53]. InstantJchem [54] was used for computing physico-chemical properties and for adding CAS number, IUPAC names, doi, references (Figure 11). Duplicate structures were removed.

### 3.2. Database Preparation

The 2D chemical structures were prepared as follows: for each molecule, the protonation state was calculated by means of the LigPrep module [55] as implemented in Schrodinger Suite ver. 11 [56,57], using additional protonated and tautomeric forms at pH 7.4. All ligands were optimized using OPLS_2005 force field and submitted to virtual screening.

### 3.3. Removal of Pan Assay Interference Compounds (PAINS) from Screening Libraries

**Figure 12:**
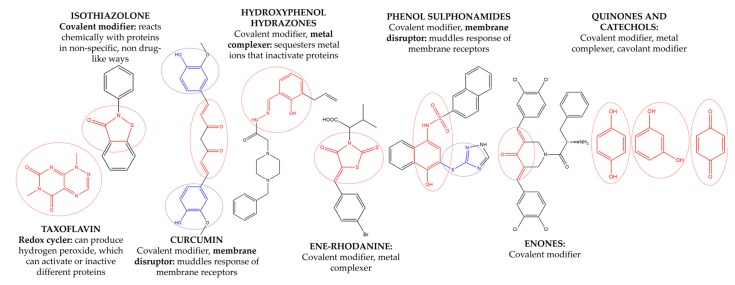
The chemical substructures of PAINS (in blue and red show reactive portions).

The discovery of new hits by in silico screening may be highly productive but can lead to false positive hits. Such compounds have been recently introduced as Pan Assays INterference compounds (PAINS) or colloidal aggregators. This can strongly influence the identification of new bioactive compounds since it can origin false activity signals [58]. For this reason, several public filters have been introduced in the last years to identify both PAINS and aggregators. Some examples are ZINC15 [59], CBLIGAND [60], FAFDRUGS3 [61] and ADVISOR [62] websites [63]. Some example of PAINS substructures are shown in Figure 12 [58]. In this study, the ZINC15 algorithm was used to detect PAINS compounds while approximately 119 compounds were deleted thus obtaining a final database of 984 hits that were submitted to docking simulations.

### 3.4. Targets Preparation and Clustering

The 3D starting coordinates of the 43 targets involved in the pathophysiological process were selected from the PDB (Table S4 of the Supplementary Materials). The selection criteria for the X-ray targets were: (a) provenance from *Homo sapiens*; (b) the presence of one co-crystallized ligand; (c) the crystallographic resolution; (d) the PDB deposition data; (e) the capability of this study’s protocol to reproduce the experimental complex using the re-docking procedure.

For each selected PDB entry, the Protein Preparation Wizard [57,64] was used to add hydrogens, to assign partial charges, to build missing atoms, side chains and loops. The resulting structures were submitted to energy optimisation using the same previously reported protocol. The co-crystallized ligands were used to generate the docking grid box and to check the prediction of the binding affinity and geometry. Therefore, for each target, the crystallograhic pose of the inhibitor complexed in each X-ray model was submitted to the docking simulation and RMSD calculation with respect to the experimental. The PDB models used for the virtual screening were clustered according to their physiopathologic role: targets involved in cancer, in neuronal degenerative diseases, in inflammatory and metabolic diseases (Figure S5 of the Supplementary Materials).

### 3.5. Docking Simulation

Docking studies were perfomed using the software Glide ver. 7.2 [65]. The binding affinity (G-Score) for each of the 984 compounds against 43 target sequences was predicted using the Glide Standard Precision (SP) protocol. Ten poses per ligand were taken into account.

### 3.6. Data Analysis

The most promising compounds were selected taking into account the G-score value. In particular, we used a cut-off equal to −8.00 Kcal/mol. Subsequent analysis was performed by matrix formulations that allowed us to identify the compounds with affinity for different targets implicated in complicated diseases (previously described).

## 4. Conclusions

From a virtual screening analysis, the current study identified compounds having good theoretical binding affinities against targets involved in complicated diseases. Indeed, chemical compounds from mushrooms provide more opportunities for finding new lead structures for medicinal chemistry possessing drug-like features. The present work study highlights the compounds extracted from *I. obliquus*, *G. lucidum* and *H. erinaceus* such as inotopyrrole, ganomycin B, hericenol A and erinacerin P respectively, as having the potential to bind anticancer targets. Other molecules, such as illudacetalic acid and pterulinic acid, extracted from omphalotus olearius, showed theoretical binding affinity against targets involved in inflammation. Cordysinin A and pterulone exhibited affinity to targets involved in neurodegenerative diseases while O-xylotocopherol versus targets implicated in metabolic disorders. Perlolyrine, an alkaloid extracted from *C. sinensis*, is reported to have good theoretical binding affinity versus relevant targets involved in the inflammatory process and in neurodegenerative diseases (confirming generic literature data) [15]. The fact that several drugs exert their effect through interaction with multiple targets, is shifting the drug discovery paradigm from the one target–one drug model to a multiple-target approach. In this context, instead of pursuing highly selective compounds for unique targets, that is, ‘single keys for specific locks,’ the goal is moving towards identifying ideal ‘master keys’ that selectively operate on a set of ‘multiple locks’ gaining access to a clinical benefit that is usually associated with a complex biological process. Of course, the ‘master key’ should not open any lock (off-target) to avoid adverse effects [66]. In the future, the bioactivities of some underutilized mushrooms could be comprehensively evaluated and a number of bioactive compounds could be suggested for in vitro and in vivo tests. Furthermore, some mushrooms possessing various health benefits could be developed into functional foods or medicines for the prevention and treatment of several chronic diseases. The construction of the chemoinformatic database has led to deepening knowledge about species that are still little explored and known. The chemical structures will be available online on a public accessible website [67,68].

## Figures and Tables

**Table 1:** Fungal components with best theoretical affinities (G-score) against multi-anticancer targets.

Name	Structure	Targets	G-Score (kcal/mol)
Inotopyrrole	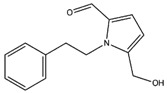	c-Met	−8.05
		VEGFR2	−8.08
		EGFR	−8.22
		MEK2	−8.09
Marasmone 21	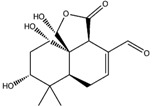	c-Met	−8.34
		MEK1	−8.02
Fascicularone A	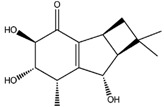	c-Met	−8.51
		SIRT1	−8.78
Enokipodin H	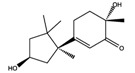	c-Met	−8.79
		GSK3β	−8.21
		SIRT1	−8.40
Confluentin	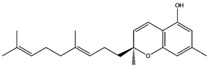	VEGFR2	−10.16
		EGFR	−10.16
		PDK1	−8.97
		SIRT1	−8.75
Blennin C	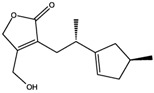	c-Met	−10.16
		MEK1	−8.97
		SIRT1	−8.75
Acromelic acid A	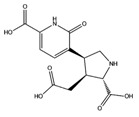	PDK1	−8.50
		CA IX	−8.22
		CA XII	−8.32
Ganomycin B	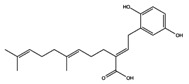	VEGFR2	−8.44
		B-RAF V600E	−8.29
		PDK1	−8.74
		SIRT1	−8.19
Atromentic acid	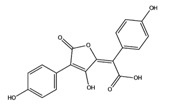	c-Met	−8.51
		VEGFR2	−9.09
		B-RAF wt	−8.5
		PDK1	−8.14
1-O-Acetyl-3-epi-illudol	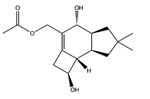	c-Met	−8.97
		MEK1	−9.37
		PDK1	−8.25
		GSK3β	−8.4
Gentianal	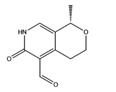	c-Met	−8.77
		VEGFR2	−8.5
		EGFR	−8.27
		B-RAF V600E	−8.08
		BMX	−8.02
Hericenone H	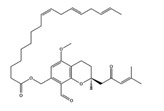	c-Met	−8.82
		EGFR	−8.48
Hericenol A	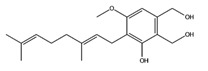	c-Met	−8.68
		MEK1	−8.00
		MEK2	−8.17
		SIRT1	−8.46
Erinacerin P	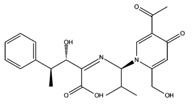	MEK1	−8.48
		SGK1	−8.06
Erinacerin A	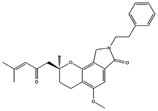	EGFR	−8.2
		B-RAF wt	−8.48
		SIRT1	−8.36
Hericenol B	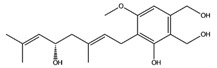	c-Met	−9.52
		EGFR	−8.41
		MEK1	−8.66
		MEK2	−8.89
		SIRT1	−9.11
Genistin	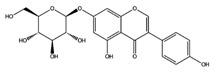	VEGFR2	−8.22
		B-RAF wt	−8.91
		MEK1	−9.52
		SGK1	−8.39
Illudinine	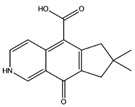	Aurora A kinase	−8.59
		c-Met	−8.89
		VEGFR2	−8.79
		B-RAF V600E	−8.9
		BMX	−8.13
		GSK3β	−8.67
		SIRT1	−8.11
Daldinal	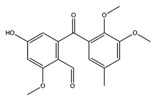	c-Met	−8.62
		VEGFR2	−8.85
		B-RAF V600E	−8.03
		PI3Kγ	−8.34
Narigin	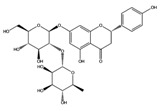	ERK1	−8.42
		B-RAF wt	−9.89
		B-RAF V600E	−9.64
		MEK1	−9.12
		BMX	−10.04
		SGK1	−8.99
(*R*)-Torosachrysone	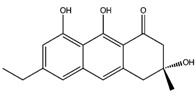	c-Met	−8.96
		VEGFR2	−8.69
		ERK1	−8.29
		MEK1	−8.53
		PI3Kα	−8.06
		BMX	−8.22
		GSK3β	−8.55
		SIRT1	−8.04

**Table 2:** Fungal components with best theoretical affinities (G-score) against multiple targets involved in the mechanism of inflammation.

Name	Structure	Target	G-Score (kcal/mol)
**Illudacetalic Acid**	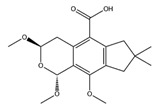	COX-2	−8.42
		Adenosine A2A R	−8.09
**Pterulinic Acid**	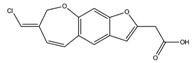	COX-1	−8.97
		Glucocorticoid R	−8.73
**Orellanine**	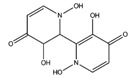	COX-1	−8.64
		COX-2	−8.85
		Glucocorticoid R	−8.39
**Bisnoryangonin**	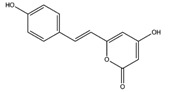	COX-2	−8.17
		Glucocorticoid R	−8.34
**1-Naphthylacetic acid**	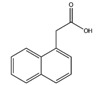	COX-1	−9.33
		COX-2	−8.33
**Pulvinic acid**	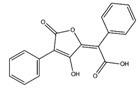	COX-2	−8.56
		Glucocorticoid R	−8.23

**Table 3:** Fungal components with best theoretical affinities (G-score) against multiple targets involved in the neurodegenerative diseases.

Name	Structure	Targets	G-Score (kcal/mol)
Erinacerin O	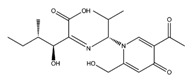	AChE	−8.70
		COMT	−8.00
Cordysinin A	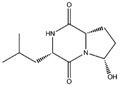	GSK3β	−8.13
		AChE	−8.47
		COX-2	−8.34
Hesperidin	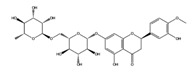	AChE	−10.67
		COMT	−9.18
		AdenosineA2A R	−9.63
Vitexin	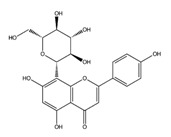	AChE	−8.93
		COMT	−8.09
Pterulone	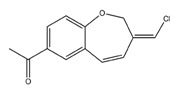	AChE	−8.38
		MAO-B	−8.16
		COX-2	−8.54
		GSK3β	−8.09

**Table 4:** Fungal components with best theoretical affinities (G-score) against multiple targets involved in the metabolic diseases.

Name	Structure	Targets	G-Score (kcal/mol)
Hericenone A	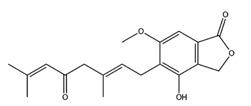	Insulin R	−8.62
		IGFR	−8.23
		PPAR-α	−8.49
		PPAR-γ	−8.81
		GSK3β	−8.01
O-xylotocopherol	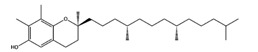	PPAR-α	−9.00
		PPAR-γ	−9.20
3,4-Dihydro-2-methyl-2-(2-oxo-4-methyl-3-pentenyl)-5-methoxy-2*H*-furo[3,4-h][1]benzopyran-9(7*H*)-one	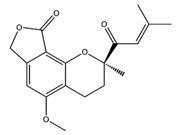	PPAR-α	−8.05
		PPAR-γ	−8.27
7-Acetyl-4-methylazulene-1-carboxylic acid	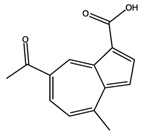	Insulin R	−8.12
		PPAR-α	−8.43
4,6-Dihydroxyisobenzofuran-1,3-dione	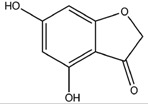	**PKA C-α**	**−8.84**
		PPAR-γ	−8.49
		GSK3β	−8.10

## Supplementary Materials

Supplementary materials are available online.
