# Supplementary material for: Chemoinformatic Database Building and in Silico Hit-Identification of Potential Multi-Targeting Bioactive Compounds Extracted from Mushroom Species
Source: Molecules. 2017 Sep 19;22(9):1571. doi: 10.3390/molecules22091571 (PMC6151421; doi:10.3390/molecules22091571)
Supplement: Supplementary file 1 [file molecules-22-01571-s001.pdf]

Supplementary Materials

Chemoinformatic Database Building and *in Silico* Hit-Identification of Multi-targeting Bioactive Compounds Extracted from Mushroom Species

Annalisa Maruca, Federica Moraca, Roberta Rocca, Fulvia Molisani, Francesca Alcaro, Maria Concetta Gidaro, Stefano Alcaro *, Giosuè Costa and Francesco Ortuso

Laboratorio di Chimica Farmaceutica, Dipartimento di Scienze della Salute, Università "Magna Græcia" di Catanzaro, Viale Europa, 88100 Catanzaro, Italy; maruca@unicz.it (A.M.); fmoraca@unicz.it (F.Mor.); rocca@unicz.it (R.R.); fulviamolisani@gmail.com (F.Mol.); francesca.alcaro@gmail.com (F.A.); mgidaro@unicz.it (M.C.G.); gcosta@unicz.it (G.C.), ortuso@unicz.it (F.O.)

* Correspondence: alcaro@unicz.it; Tel.: +39-0961-369-4197

Table S1. Re-docking experiments details with the relative co-crystallized ligand of each PDB model.

Anticancer activity			
**Target**	**PDB code**	**Co-crystallized ligand**	**G-score (kcal/mol)**
c-Met	2WGJ	Crizotinib	−12.03
MEK1	4ARK	2-([3*R*-3,4-Dihydroxy-butyl]oxy)-4-fluoro-6- [(2-fluoro-4-iodophenyl)-amino]benzamide	−8.64
SIRT1	4I5I	(6*S*)-2-Chloro-5,6,7,8,9,10-hexahydrocyclohepta[b]indole-6-carboxamide	−10.36
MEK2	1S9I	5-[3,4-Difluoro-2-(2-fluoro-4-iodophenylamino)-phenyl]-[1,3,4]oxadiazol-2-yl)-(2-morpholin-4-yl-ethyl)-amine	−8.61
SGK1	3HDM	4-(5-Phenyl-1*H*-pyrrolo[2,3-b]pyridin-3-yl)benzoic acid	−9.77
VEGFR2	3VHE	Pyrrolopyrimidine derivative	−13.9
EGFR	3POZ	Tak-285	−13.89
B-RAF V600E	3OG7	Vemurafenib	−12.24
PDK1	3NAX	1-(3,4-Difluorobenzyl)-2-oxo-N-{(1R)-2-[(2-oxo-2,3-dihydro-1*H*-benzimidazol-5-yl)oxy]- 1-phenylethyl}-1,2-dihydro-pyridine-3-carboxamide	−18.82
**Anti-inflammatory activity**			
**Target**	**PDB code**	**Co-crystallized ligand**	**G-score (kcal/mol)**
COX-2	5IKR	Mefenamic Acid	−10.04
Adenosine A2A R	3RFM	Caffeine	−8.09
COX-1	1Q4G	α-Methyl-4-biphenylacetic acid	−10.73
Glucocorticoid	1M2Z	Dexamethasone	−12.35

Table S1. *Cont.*

Neurodegenerative activity			
**Target**	**PDB code**	**Co-crystallized ligand**	**G-score (kcal/mol)**
GSK3b	4ACC	5-[3,4-Difluoro-2-(2-fluoro-4-iodoanilino)phenyl]-N-(2-morpholin-4-ylethyl)-1,3,4-oxadiazol-2-amine	−9.15
AChE	4EY7	Donepezil	−12.79
MAO-B	2V5Z	Safinamide	−9.12
**Metabolic disease activity**			
**Target**	**PDB code**	**Co-crystallized ligand**	**G-score (kcal/mol)**
PPAR-α	3VI8	APHM13	−14.51
PPAR-γ	2PRG	Rosiglitazone	−12.46

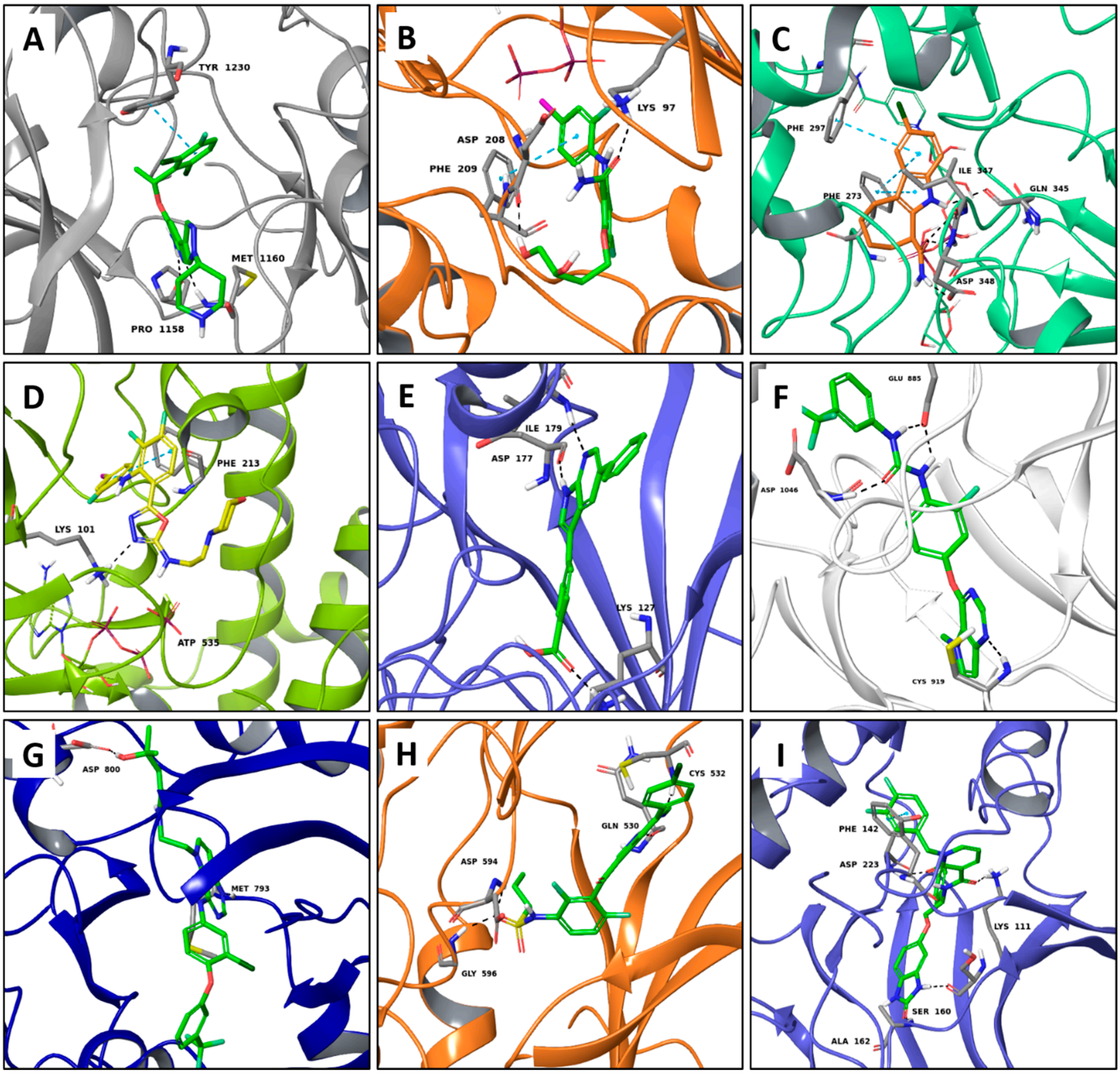

Figure S1. Re-docked best pose of the relative co-crystallized ligands in the anticancer PDB models: (**A**) c-Met (PDB code: 2WGJ); (**B**) MEK1 (PDB code: 4ARK); (**C**) SIRT1 (PDB code: 4I5I); (**D**) MEK2 (PDB code: 1S9I); (**E**) SGK1 (PDB code: 3HDM); (**F**) VEGFR2 (PDB code: 3VHE); (**G**) EGFR (PDB code: 3POZ); (**H**) B-RAF V600E (PDB code: 3OG7); (**I**) PDK1 (PDB code: 3NAX).

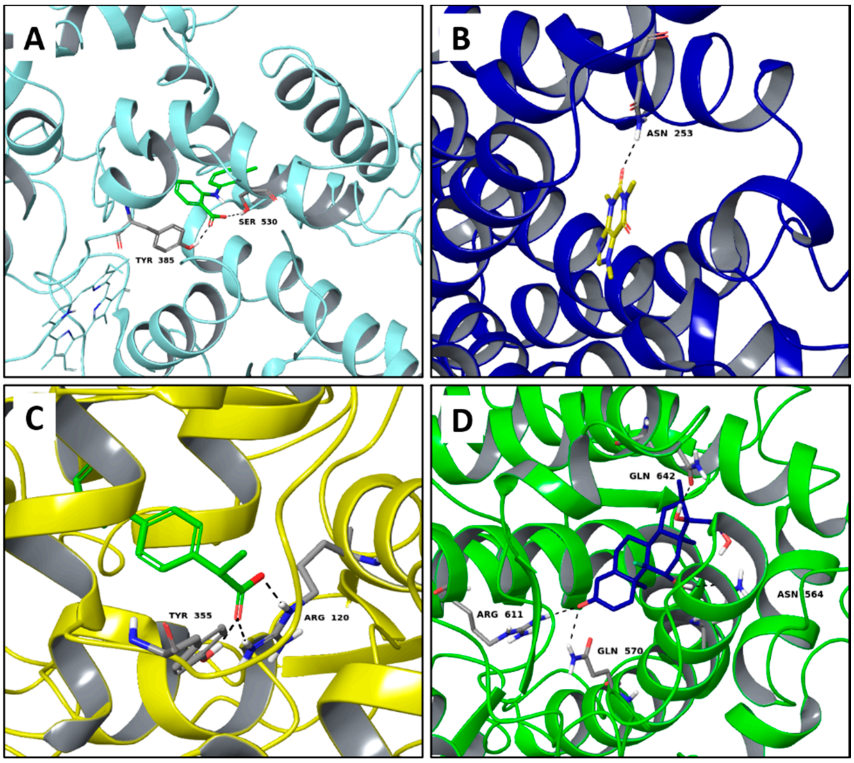

Figure S2. Re-docked best pose of the relative co-crystallized ligands in the anti-inflammatory PDB models: (**A**) COX-2 (PDB code: 5IKR); (**B**) Adenosine A2A R (PDB code: 3RFM); (**C**) COX-1 (PDB code: 1Q4G); (**D**) Glucocorticoid R (PDB code: 1M2Z).

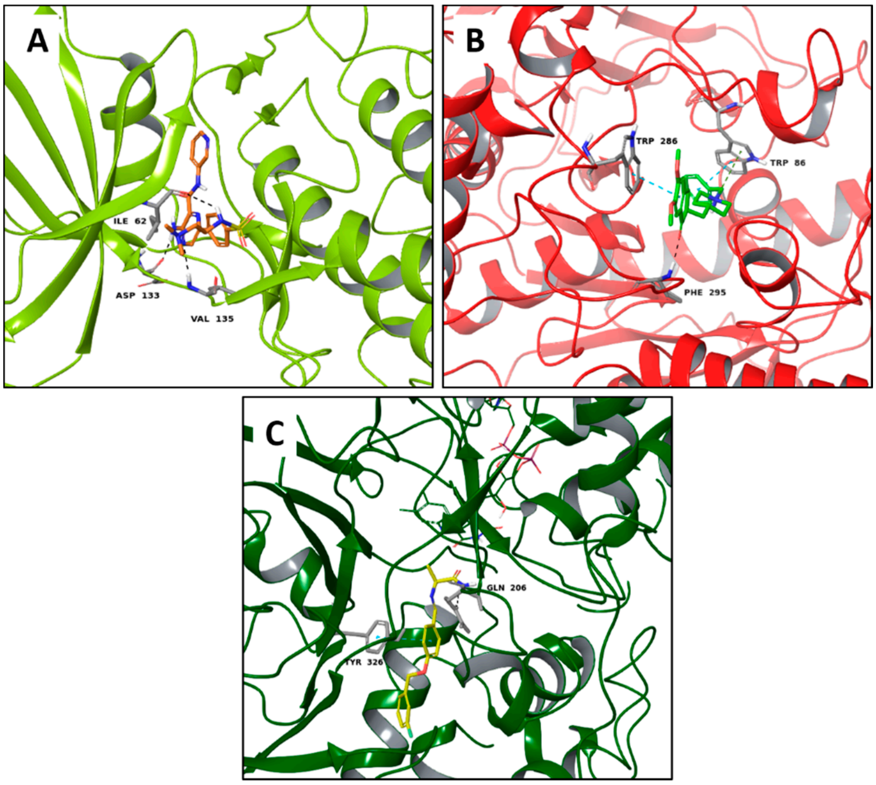

Figure S3. Re-docked best pose of the relative co-crystallized ligands in the main targets involved in the neurodegenerative diseases: (**A**) GSK3β (PDB code: 4ACC); (**B**) AChE (PDB code: 4EY7); (**C**) MAO-B (PDB code: 2V5Z).

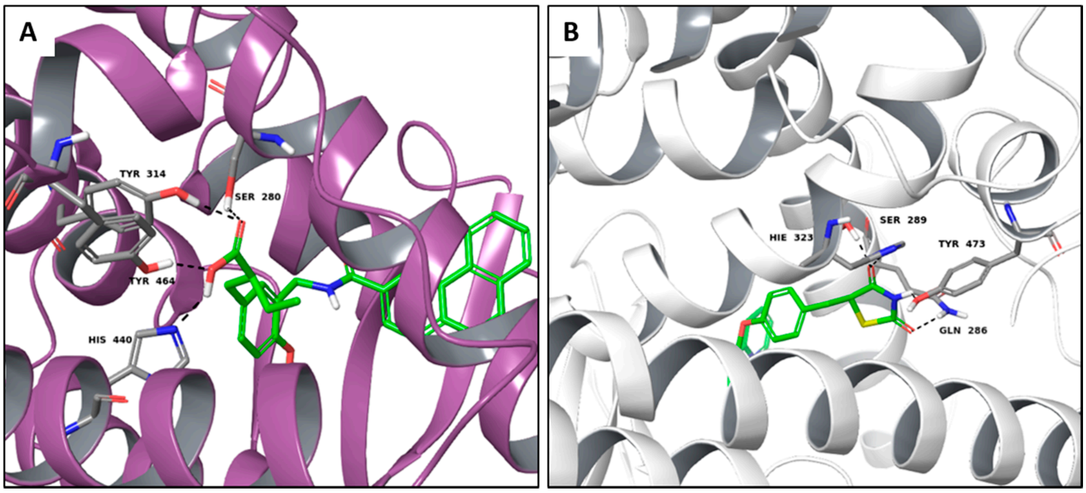

Figure S4. Re-docked best pose of the relative co-crystallized ligands in the main targets involved in the metabolic diseases: (**A**) PPAR-α (PDB code: 3VI8); (**B**) PPAR-γ (PDB code: 2PRG).

Table S2. Non-selective MTAs.

Name	2D Structure	Targets	G-score (kcal/mol)
**Fuscoprine**	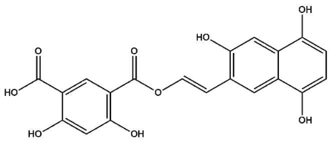	Aurora B kinase	-8.76
		c-Met	-9.48
		VEGFR2	-10.37
		EGFR	-10.43
		ERK1	-8.56
		B-RAF wt	-10.05
		B-RAF V600E	-9.1
		BMX	-8.74
		Akt	-8.99
		CDK2	-9.2
		SGK1	-9.36
		CA IX	-8.2
		SIRT1	-8.58
		PKA C-α	-9.7
		PPAR-α	-8.98
		AChE	-9.97
		BuChE	-8.83
		MAO-B	-8.84
		AdenosneA2A R	-8.1
		Glucocorticoid R	-9.72
**Ganomycin A**	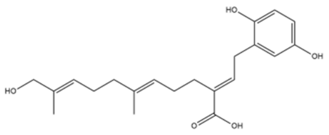	c-Met	-8.65
		VEGFR2	-8.65
		B-RAF wt	-8.17
		B-RAF V600E	-8.41
		MEK1	-9.24
		PDK1	-9.36
		IGF-1R	-8.12
		PPAR-γ	-9.09
		COX-1	-8.27
		AChE	-8.54

Table S2. *Cont.*

**Narigenin**	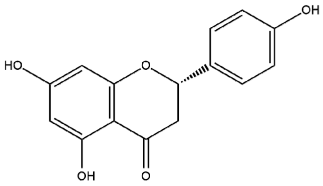	**c-Met<br>VEGFR2<br>EGFR<br>EGFR V948R<br>ERK2<br>B-RAF wt<br>PI3Kα<br>PI3Kγ<br>BMX<br>CDK2<br>IGFR<br>PPAR-γ<br>AChE<br>MAO-A<br>COMT<br>COX-1<br>COX-2<br>AdenosineA2A R<br>Glucocorticoid R**	**-8.2<br>-9.44<br>-8.23<br>-8.2<br>-8.55<br>-9.15<br>-8.25<br>-8.14<br>-8.52<br>-9.02<br>-8.72<br>-8.42<br>-9.03<br>-8.18<br>-8.67<br>-8.22<br>-8.86<br>-8.97<br>-8.74**
**Enokipodin G**	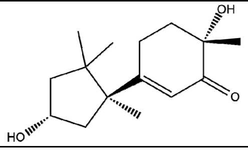	c-Met<br>GSK3β<br>PPAR-α<br>Glucocorticoid R	-8.64<br>-8.08<br>-8.03<br>-9.74
**Enokipodin H**	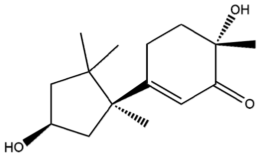	c-Met<br>GSK3β<br>SIRT1<br>PPAR-α<br>Glucocorticoid R	-8.79<br>-8.21<br>-8.4<br>-8.11<br>-8.59
**Hericenol C**	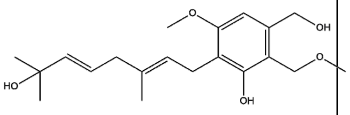	MEK1<br>SIRT1<br>PPAR-α<br>PPAR-γ<br>AChE<br>Glucocorticoid R	-8.37<br>-8.34<br>-8.34<br>-8.33<br>-8.49<br>-8.13
**Acetylhispidin**	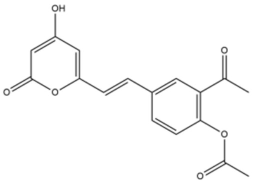	c-Met<br>VEGFR2<br>EGFR<br>B-RAF wt<br>PPAR-α<br>PPAR-γ<br>AChE<br>MAO-B<br>Glucocorticoid R	-8.9<br>-9.59<br>-8.57<br>-8.28<br>-8.87<br>-8.6<br>-8.22<br>-8.2<br>-8.32

Table S2. *Cont.*

**Pterulone B**	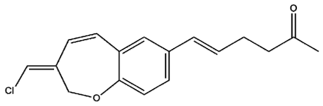	**VEGFR2**<br>**MEK1**<br>**PI3Kγ**<br>**SIRT1**<br>**PPAR-α**<br>**PPAR-γ**<br>**MAO-A**<br>**MAO-B**<br>**COX-1**<br>**Glucocorticoid R**	**-8.45**<br>**-8.77**<br>**-8.1**<br>**-8.31**<br>**-8.36**<br>**-8.08**<br>**-8.28**<br>**-8.7**<br>**-8.4**<br>**-9.19**
**Cordyceamide B**	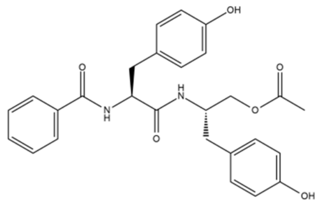	c-Met<br>EGFR<br>PI3Kγ<br>PDK1<br>PPAR-α<br>PPAR-γ<br>AChE<br>BuChE<br>IL-17A<br>AdenosineA2A R	-9.21<br>-8.41<br>-9.81<br>-11.09<br>-9.13<br>-10.12<br>-10.05<br>-9.93<br>-10.27<br>-8.69
**Aurantiamide Acetate**	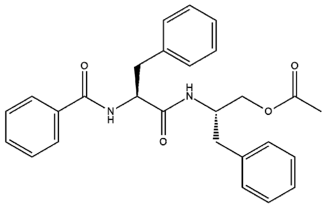	Aurora B kinase<br>c-Met<br>EGFR<br>PDK1<br>PPAR-α<br>PPAR-γ<br>AChE<br>BuChE<br>IL-17A<br>AdenosineA2A R	-8.13<br>-8.09<br>-8.24<br>-10.6<br>-10.24<br>-8.45<br>-9.55<br>-8.83<br>-9.95<br>-9.2
**Glycitein**	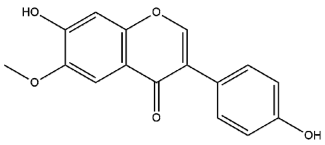	VEGFR<br>B-RAF wt<br>B-RAF V600E<br>PDK1<br>SIRT1<br>PPAR-γ<br>AChE<br>COMT<br>COX-1<br>AdenosineA2A R<br>Glucocorticoid R<br>VEGFR2<br>EGFR	-9.79<br>-8.11<br>-8.18<br>-8.23<br>-8.4<br>-8.14<br>-8.39<br>-8.05<br>-8.47<br>-8.39<br>-9.03<br>-9.3<br>-8.56
**Erinacerin T**	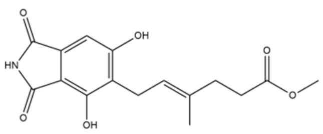	B-RAF wt<br>B-RAF V600E<br>CDK2<br>SIRT1<br>PKA C-α<br>PPAR-α<br>PPAR-γ<br>AChE<br>Glucocorticoid R	-8.7<br>-9.59<br>-8.15<br>-9.17<br>-9.57<br>-9.06<br>-10.34<br>-8.57<br>-8.91

Table S2. *Cont.*

**Cordysinin C**	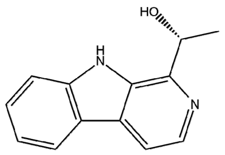	**MEK1**<br>**GSK3β**<br>**CDK2**<br>**SIRT1**<br>**Insulin R**<br>**IGF-1R**<br>**PPAR-α**<br>**MAO-A**<br>**COMT**<br>**COX-1**<br>**Glucocorticoid R**	**-8.95**<br>**-8.09**<br>**-8.51**<br>**-8.77**<br>**-8.28**<br>**-8.92**<br>**-8.29**<br>**-8.61**<br>**-8.59**<br>**-8.35**<br>**-9.24**
**Cordyceamide A**	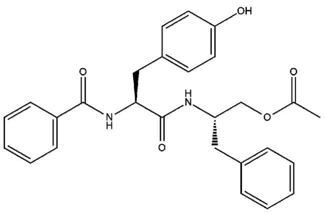	c-Met<br>EGFR<br>MEK1<br>PDK1<br>IGF-1R<br>PPAR-α<br>PPAR-γ<br>AChE<br>BuChE<br>IL-17A<br>AdenosineA2A R	-9.22<br>-8.64<br>-9.42<br>-10.26<br>-8.89<br>-9.86<br>-8.79<br>-10.23<br>-9.51<br>-10.54<br>-8.05
**Erinacerin S**	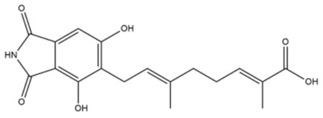	c-Met<br>VEGFR2<br>B-RAF V600E<br>PDK1<br>CDK2<br>SIRT1<br>PKA C-α<br>PPAR-α<br>PPAR-γ<br>AChE<br>AdenosineA2A	-8.28<br>-8.46<br>-8.29<br>-8.98<br>-8.27<br>-9.49<br>-9.81<br>-9.85<br>-10.65<br>-8.77<br>-8.54
**Cordysinin D**	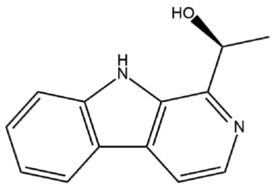	ERK2<br>P38 MAP kinase<br>PDK1<br>GSK3β<br>CDK2<br>SIRT1<br>Insulin R<br>PPAR-α<br>MAO-A<br>COMT<br>COX-1<br>Glucocorticoid R	-8.24<br>-8.1<br>-8.24<br>-8.64<br>-8.0<br>-8.51<br>-8.5<br>-8.18<br>-8.25<br>-8.62<br>-8.66<br>-8.76

Table S2. *Cont.*

**Riboflavin**	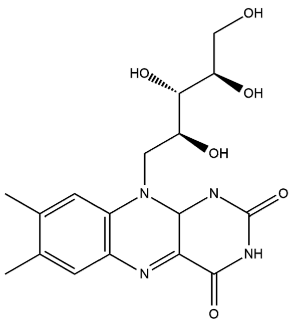	**EGFR V948R**<br>**B-RAF wt**<br>**MEK1**<br>**PI3Kγ**<br>**GSK3β**<br>**CDK2**<br>**Insulin R**<br>**PPAR-γ**<br>**AChE**<br>**BuChE**<br>**COMT**<br>**AdenosineA2A R**<br>**Glucocorticoid R**	**-8.62**<br>**-9.55**<br>**-8.63**<br>**-9.32**<br>**-8.67**<br>**-8.06**<br>**-8.56**<br>**-8.14**<br>**-8.75**<br>**-8.32**<br>**-9.1**<br>**-8.0**<br>**-8.74**
**Erinacerin B**	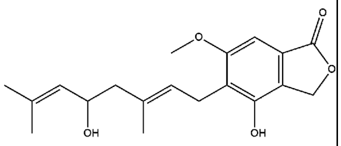	c-Met<br>VEGFR2<br>EGFR<br>B-RAF V600E<br>MEK1<br>SIRT1<br>Insulin R<br>PKA C-α<br>PPAR-α<br>PPAR-γ<br>AChE<br>MAO-A<br>AdenosineA2A R<br>Glucocorticoid R	-8.44<br>-8.26<br>-8.35<br>-8.94<br>-8.03<br>-8.86<br>-8.94<br>9.17<br>-8.86<br>-9.55<br>-8.59<br>-8.17<br>-8.02<br>-9.74
**Genistein**	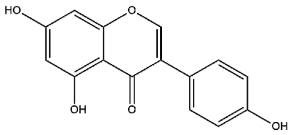	c-Met<br>VEGFR2<br>EGFR<br>ERK2<br>B-RAF wt<br>MEK1<br>SIRT1<br>IGF-R1<br>PPAR-γ<br>AChE<br>COMT<br>COX-1<br>COX-2<br>AdenosineA2A R	-8.19<br>-10.36<br>-8.26<br>-8.82<br>-8.21<br>-8.04<br>-8.01<br>-8.21<br>-8.01<br>-8.26<br>-8.45<br>-9.05<br>-8.54<br>-8.76
**Apigenin-7-glucoside**	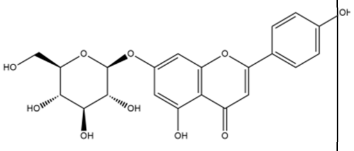	VEGFR2<br>EGFR<br>ERK1<br>B-RAF wt<br>MEK1<br>PI3Kγ<br>PDK1<br>BMX<br>CDK2<br>Insulin R<br>PPAR-α<br>PPAR-γ<br>AChE<br>COMT<br>AdenosineA2A R	-8.69<br>-9.73<br>-8.13<br>-8.99<br>-9.24<br>-8.51<br>-8.02<br>-9.48<br>-9.22<br>-8.43<br>-8.18<br>-9.22<br>-9.87<br>-8.25<br>-9.76

Table S2. *Cont.*

**Liquiritigenin**	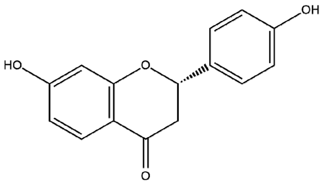	**c-Met**	**-8.43**
		**VEGFR2**	**-10.26**
		**EGFR**	**-8.13**
		**EGFR V948R**	**-8**
		**ERK1**	**-8.4**
		**B-RAF wt**	**-9.29**
		**B-RAF V600E**	**-8.06**
		**MEK1**	**-8.65**
		**PI3K-α**	**-8.1**
		**PI3K-γ**	**-8.0**
		**BMX**	**-8.42**
		**CDK2**	**-9.06**
		**SGK1**	**-8.3**
		**SIRT1**	**-8.10**
		**AChE**	**-8.93**
		**MAO-A**	**-8.86**
		**COMT**	**-8.26**
		**COX-1**	**-8.39**
		**COX-2**	**-9.32**
		**AdenosineA2A R**	**-8.74**
		**Glucocorticoid R**	**-9.58**
**Calycosin**	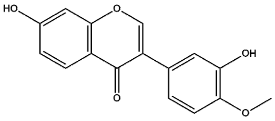	VEGFR2	-9.64
		EGFR	-8.43
		ERK2	-8.25
		B-RAF wt	-8.19
		BMX	-8.29
		CDK2	-8.81
		SIRT1	-8.61
		Insulin R	-8.01
		IGF-1R	-8.12
		PPAR-α	-8.70
		PPAR-γ	-8.20
		AChE	-8.83
		COX-1	-8.64
		COX-2	-8.00
		AdenosineA2A R	-8.06
		Glucocorticoid R	-9.87

Table S3. Classification of edible and not edible mushrooms with species and family.

**Edible mushrooms**		**Toxic mushrooms**	
**Species**	**Family**	**Species**	**Family**
Agaricus arvensis	Agaricaceae	*Agaricus xanthodermum*	Agaricaceae
Agaricus bisporus	"	*Chlorophyllum rachodes*	"
Agaricus bitorquis	"	*Lepiota brunneoincarnata*	"
Agaricus campestris	"	*Lepiota helveola*	"
Agaricus subrufescens	"	*Lepiota subincarnata*	"
Macrolepiota procera	"	*Amanita aminoaliphatica*	Amanitaceae
Albatrellus pescaprae	Albatrellaceae	*Amanita junquillea*	"
Amanita caesarea	Amanitaceae	*Amanita muscaria*	"
Amanita rubescens	"	*Amanita ovoidea*	"

Table S3. *Cont.*

Volvariella volvacea	Amanitaceae	*Amanita pantherina*	Amanitaceae
Auricularia auricula-judae	Auriculariaceae	*Amanita phalloides*	"
Boletus aereus	Boletaceae	*Amanita proxima*	"
Boletus appendiculatus	"	*Amanita smithiana*	"
Boletus edulis	"	*Amanita verna*	"
Boletus erythropus	"	*Amanita virosa*	"
Boletus luridus	"	*Panaeolus cyanescens*	Bolbitiaceae
Boletus pinicola	"	*Panaeolus semiovatus*	"
Boletus regius	"	*Boletus pulchrotinctus*	Boletaceae
Boletus reticulatus	"	*Boletus rhodoxanthus*	"
Boletus subappendiculatus	"	*Boletus rubrosanguineus*	"
Leccinum aurantiacum	"	*Boletus satanas*	"
Leccinum scabrum	"	*Ramaria pallida*	Clavariaceae
Xerocomus badius	"	*Coprinus atramentarius*	Coprinaceae
Cantharellus cibarius	Cantharellaceae	*Cordyceps sinensis*	Cordycipitaceae
Cantharellus lutescens	"	*Cortinarius orellanoides*	Cortinariaceae
Craterellus cornucopioides	"	*Cortinarius orellanus*	"
Coprinus comatus	Coprinaceae	*Galerina autumnalis*	"
Fistulina hepatica	Fistulinaceae	*Galerina marginata*	"
Ramaria botrytis	Clavariaceae	*Gymnopilus spectabilis*	Crepidotaceae
Hericium erinaceus	Hericiaceae	*Cudonia circinans*	Cudoniaceae
Hydnum repandum	Hydnaceae	*Gyromitra esculenta*	Discinaceae
Hydnum rufescens	"	*Gyromitra fastigiata*	"
Hygrophorus hypothejus	Hygrophoraceae	*Gyromitra gigas*	"
Hygrophorus marzuolus	"	*Gyromitra infula*	"
Hygrophorus pudorinus	"	*Entoloma nidorosum*	Entolomataceae
Hygrophorus russula	"	*Entoloma sinuatum*	"
Marasmius oreades	Marasmiaceae	*Ganoderma lucidum*	Ganodermataceae
Flammulina velutipes	"	*Helvella crispa*	Helvellaceae
Grifola frondosa	Meripilaceae	*Inonotus obliquus*	Hymenochaetaceae
Morchella costata	Morchellaceae	*Inocybe bongardii*	Inocybaceae
Morchella elata	"	*Inocybe erubescens*	"
Morchella esculenta	"	*Inocybe geophylla*	"
Lentinula edodes	Omphalotaceae	*Inocybe rimosa*	Cortinariaceae
Armillaria mellea	Physalacriaceae	*Mycena inclinata*	Marasmiaceae
Pisolithus arhizus	Pisolithaceae	*Mycena pelianthina*	"
Pleurotus ostreatus	Pleurotaceae	*Mycena pura*	"
Laetiporus sulphureus	Polyporaceae	*Mycena rosea*	"
Polyporus umbellatus	"	*Omphalotus illudens*	Omphalotaceae
Lactarius deliciosus	Russulaceae	*Omphalotus olearius*	"
Lactarius salmonicolor	"	*Paxillus involutus*	Paxillaceae
Lactarius sanguifluus	"	*Paxillus rubicundulus*	"
Lactarius semisanguifluus	"	*Lenzites betulina*	Polyporaceae
Russula aurea	"	*Trametes versicolor*	"
Russula chloroides	"	*Psathyrella candolleana*	Psathyrellaceae
Russula cyanoxantha	"	*Psathyrella conopilus*	"
Russula delica	"	*Ramaria formosa*	Ramariaceae
Russula vesca	"	*Ramaria fumigata*	"
Russula virescens	"	*Russula emetica*	Russulaceae
Stropharia rugosoannulata	Strophariaceae	*Scleroderma geaster*	Sclerodermataceae
Suillus bellini	Suillaceae	*Hypholoma sublateritium*	Strophariaceae
Suillus bellinii	"	*Psilocybe coprophila*	
Suillus granulatus	"	*Psilocybe cyanescens*	"
Suillus luteus	"	*Psilocybe merdaria*	"
Tremella fuciformis	Tremellaceae	*Psilocybe muscorum*	"

Table S3. *Cont.*

Clitocybe geotropa	Tricholomataceae	*Psilocybe semilanceata*	Strophariaceae
Leucopaxillus giganteus	"	*Psilocybe serbica*	"
Lepista inversa	"	*Stropharia aeruginosa*	"
Lepista nuda	"	*Stropharia semiglobata*	"
Lepista sordida	"	*Clitocybe acromelalga*	Tricholomataceae
Lyophyllum fumosum	"	*Clitocybe amoenolens*	"
Tricholoma columbetta	"	*Clitocybe candicans*	"
Tricholoma imbricatum	"	*Clitocybe cerussata*	"
Tricholoma portentosum	"	*Clitocybe dealbata*	"
Tricholoma stans	"	*Clitocybe nebularis*	"
Tricholoma terreum	"	*Clitocybe phyllophila*	"
		Clitocybe rivulosa	"
		Tricholoma auratum	"
		Tricholoma equestre	"
		Tricholoma josserandii	"
		Tricholoma pardinum	"
		Tricholoma saponaceum	"
		Tricholoma sciodes	"
		Tricholoma virgatum	"

Table S4. Abbreviations of the 43 targets, selected for structure-based virtual screening studies, with the relative PDB accession code and resolution of X-ray model in Å, RMSD value after re-docking simulation between the experimental pose of the inhibitor into the X-ray and the best pose generated by our docking protocol and G-score.

**Target**	**PDB models**	**Resolution(Å)**	**RMSD (Å)**	**G-score (Kcal/mol)**
Akt Kinase	4GV1	1.49	1.06	-11.63
Aurora A kinase	2X81	2.91	0.47	-9.38
Aurora B kinase	2VRX	1.86	0.74	-10.18
BMX	3SXR	2.4	0.89	-11.29
B-Raf kinase	2FB8	2.9	0.68	-10.95
B-Raf Kinase V600E	3OG7	2.45	1.62	-12.24
c-Met kinase	2WGJ	1.2	0.85	-12.03
CDK2	4KD1	1.7	1.39	-11.61
EGFR Kinase	3POZ	1.5	0.89	-13.89
EGFR Kinase V948R	4I22	1.71	1.01	-8.71
ERK1	2ZOQ	2.39	0.37	-9.26
ERK2	4ZZN	1.45	0.58	-12.41
GSK3β	4ACC	2.21	1.20	-9.15
P38 MAP Kinase	3GCP	2.25	0.99	-12.06
MEK1	4ARK	2.6	0.71	-8.64
MEK2	1S9I	3.2	0.38	-8.61
PDK1	3NAX	1.75	0.34	-18.82
PI3Kα	5UBR	2.4	0.92	-10.48
PI3Kγ	3DBS	2.8	0.78	-9.34
SGK1	3HDM	2.6	0.68	-9.77
VEGFR2	3VHE	1.55	0.34	-13.90
SIRT1	4I5I	2.5	0.19	-10.36
5LOX	3V99	2.25	1.51	-4.54

Table S4. *Cont.*

COX-1	1Q4G	2.0	0.27	-10.73
COX-2	5IKR	2.34	0.36	-10,04
IL-17A	5HI5	1.8	0.33	-13.67
Adenosine A2A R	3RFM	3.6	0.69	-8.09
Glucocorticoid R	1M2Z	2.5	0.32	-12.35
DPP-4	4LKO	2.43	0.92	-6.11
IGF-1R	3I81	2.08	0.32	-10.07
Insulin R	5HHW	1.79	0.31	-12.48
PKA C-α	3POO	1.6	0.23	-12.35
HMG-CoA reductase	1HWL	2.1	0.81	-7.06
PPAR-α	3VI8	1.75	0.23	-14.51
PPAR-γ	2PRG	2.3	0.67	-12.46
AChE	4EY7	2.35	0.20	-12.79
BACE-1	5HU1	1.5	0.22	-6.6
BuChE	1P0M	2.38	Not ligand	
COMT	5LSA	1.5	0.23	-8.53
MAO-A	2Z5X	2.2	0.83	-7.675
MAO-B	2V5Z	1.6	0.58	-9.12
CA IX	5FL4	1.82	1.18	-5.67
CA XII	4WW8	1.42	1.51	-4.95

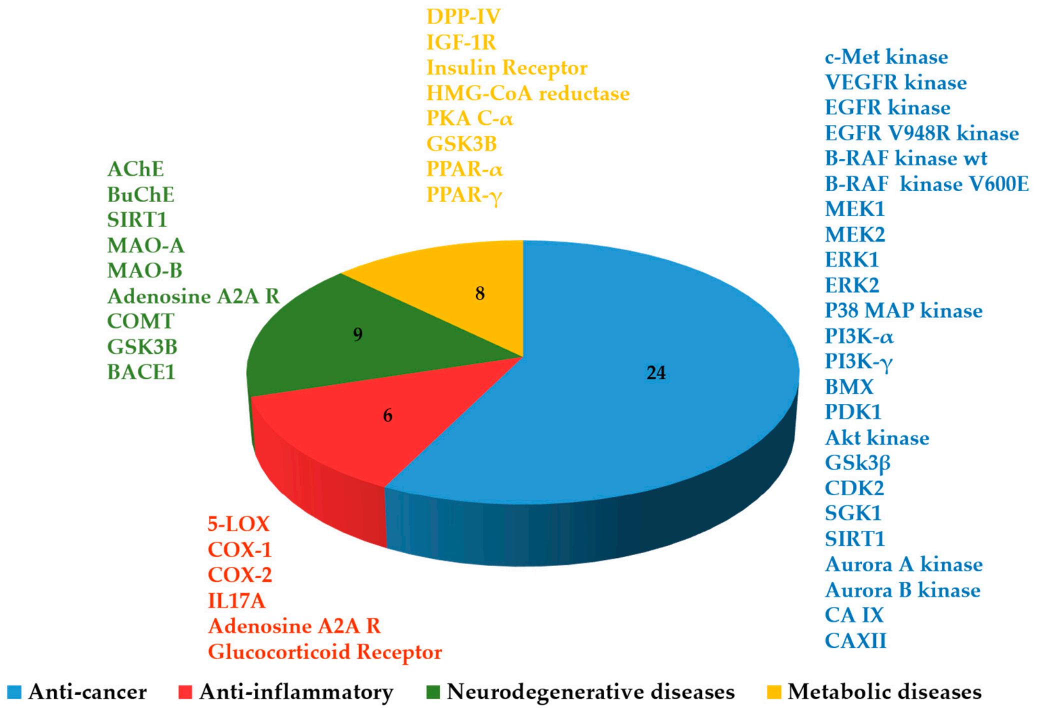

Figure S5. Clustering of biological targets.
